# Cortical Visual Performance Test Setup for Parkinson's Disease Based on Motion Blur Orientation

**DOI:** 10.1155/2019/3247608

**Published:** 2019-02-03

**Authors:** M. Erdem Isenkul

**Affiliations:** Department of Computer Engineering, Istanbul University-Cerrahpasa, 34320 Avcilar, Istanbul, Turkey

## Abstract

Studies on Parkinson's disease (PD) are becoming very popular on multidisciplinary platforms. The development of predictable telemonitored early detection models has become closely related to many different research areas. The aim of this article is to develop a visual performance test that can examine the effects of Parkinson's disease on the visual cortex, which can be a subtitle scoring test in UPDRS. However, instead of showing random images and asking for discrepancies between them, it is expected that the questions to be asked to patients should be provable in the existing cortex models, should be deduced between the images, and produce a reference threshold value to compare with the practical results. In a developed test, horizontal and vertical motion blur orientation was applied to natural image samples, and then neural outputs were produced by representing three (original-horizontal-vertical) image groups with the Layer 4 (L4) cortex model. This image representation is then compared with a filtering model which is very similar to thalamus' functionality. Thus, the linear problem-solving performance of the L4 cortex model is also addressed in the study. According to the obtained classification results, the L4 model produces high-performance success rates compared to the thalamic model, which shows the adaptation power of the visual cortex on the image pattern differences. In future studies, developed motion-based visual tests are planned to be applied to PD patient groups/controls, and their performances with mathematical threshold values will be examined.

## 1. Introduction

Parkinson's disease (PD) is a problem of progressive neural degeneration. As a result of the death of dopaminergic neurons, a great deal of negative effects occurs in some regions of the brain. PD affects different cortex areas at the same time, causing different symptoms to occur in patients. Disturbances in the cortex, especially where motor functions are regulated, affect the daily lives of patients in a negative way. Studies in the literature are mostly based on the effects of PD on motor cortex [1]. However, in recent years, with the development of technology and the dissemination of literature studies on the brain into multidisciplinary fields, the effects of PD in the previously known theoretically known but not yet studied cortical areas have begun to be examined (sensation [2, 3], perception [4], sleep [5, 6], and emotional functioning [7]).

One of these studies is the effect of PD on the visual cortex. Studies in the literature have reported that PD patients have problems in spatial perception [8, 9], spatial contrast sensitivity [10, 11], color discrimination [12, 13], and visuospatial problem solving [14] in daily life. However, there is no objective visual test to examine the patients' visual cortex health. With the development of visual tests, the visual cortex diagnostic stage will be completed. This is very important for the early detection and monitoring of disease processes.

A scoring that will be developed with a vision-based test as in UPDRS is the primary goal of this study. Furthermore, this test can be used not only for PD but also for other neurological disorders that have not yet been detected on visual acuity. For this purpose, in order to reach the gold standard, the test must be both meaningful on the human side and matched with the mathematical models of some substructures of the generally accepted visual cortex in the literature.

The threshold values or score values determined in theory will be used in questioning and problem diversification in the optimizations of the tests and will also form the basis for the determination of this distribution of controls and PD patients, which is the next step in the future studies. In this study, the theoretical foundations of these developed tests are laid out and the performance of the Layer 4 (L4) mathematical model in the primary visual cortex (V1) on the developed visual test problem is presented.

This paper is organized as follows: in Section 2, human visual processing and models are described. Sections 3 and 4 summarize the mathematical background of thalamic and cortical image representations.  Section 5 describes the dataset.  Section 6 gives brief information of the motion blur orientation method.  Section 7 presents the experimental results. We present the conclusions and discussions in Sections 8 and 9.

## 2. Human Visual Processing and Models

The human vision system processes many different retinal images and adapts to the similarities and differences between these images. In addition, adaptive neurons in the visual cortex learn that object by extracting many different features from image patterns. The studies that model this physiological learning process constitute a significant part of today's computational neuroscience studies.

V1 is the most commonly studied structure among the other visual fields and is located at the back of the occipital lobe. It is also the cortical field of vision in which the filtered information known as the lateral geniculate nucleus (LGN) is first processed. V1 is especially specialized on static and moving objects, and produces quite powerful outputs to be used in pattern recognition.

The V1 field learns a number of nonlinear interactions using inputs from sublayers and thalamus itself. The inputs used here are also in continuous interaction with the inputs to the top layers. The output is produced as a result of this interaction. The V1 area is composed of 6 different layers (labelled 1–6), and each layer is functionally different from each other. Layer 4 (L4) is the first layer of the visual cortex. According to the hypothesis, some problem linearization processes are applied in this layer. L4 converts incoming inputs into a new form and forwards them to Layer 2/3 (L2/3) in the same cortex area for further processing. The output of the L2/3 layer is sent to the L4 layers in the upper areas (V2), where the processing is performed at a higher level [15, 16].

Although the function of L4 is not fully understood, there are different suggestions in this regard in the literature; redundancy reduction [17]; input-output information maximization [18, 19]; the preservation of the spatial relationship between inputs [20]; effective distributed coding [21–23]; and problem linearization [24]. The performance of the L4 models developed for these purposes is measured by comparing the invasive measurements with the electrophysiological methods. However, due to the complex nature of the method and the cost of the method, the capacity measurement of the model becomes very difficult.

In the proposed study, the visual test performances of the Somers thalamus model [25] which based on filtering the images through thalamus and the Favorov L4 model [24] were compared. In this respect, machine learning-based classification problems have been developed which will be able to measure visual cortex layer models and produce outputs according to different weight optimization values of models in this context.

## 3. Thalamic Image Representation

**Figure 1:**
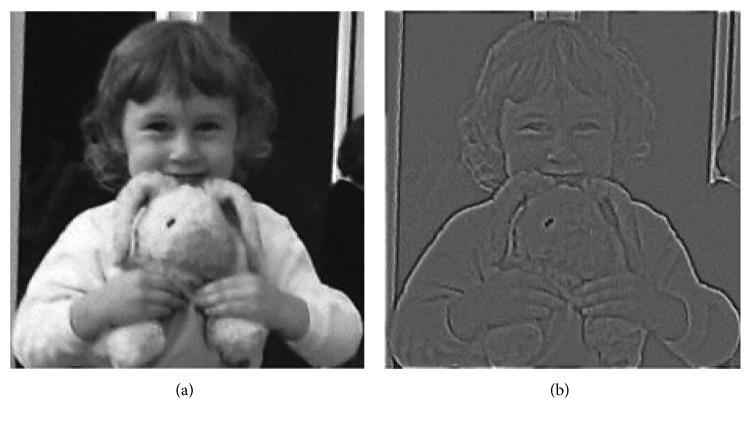
An example of thalamic representation: input image of cornea (a) and neural output image of thalamus (b).

LGN-like neurons were modelled using the retinal/LGN model [25] in order to generate realistic visual afferent inputs to L4 from LGN cells in the thalamus. The LGN layer consists of 91 ON-center and 91 OFF-center receptive fields intersected on the top. The RF profile is derived by calculating two-dimensional Gaussian differences between “central” and “surround” (1), and the ON-center and OFF-center activities of the corresponding neuron are calculated by multiplying this profile by the gray scaled pixel value ((2) and (3)):

(1) $$\begin{array}{c} {\text{RF}}_{xy}=\left(\left(\frac{1}{2\pi \sigma_{\text{center}}^{2}}\right)\exp\left(-\frac{D_{xy}^{2}}{2\sigma_{\text{center}}^{2}}\right)\right)-\left(\left(\frac{1}{2\pi \sigma_{\text{surr}}^{2}}\right)\exp\left(-\frac{D_{xy}^{2}}{2\sigma_{\text{surr}}^{2}}\right)\right), \end{array}$$

(2) $$\begin{array}{c} {\text{LGN}}_{\text{ON}}={\left[0.1+\underset{x}{\sum }\underset{y}{\sum }{\text{RF}}_{xy}{\text{PI}}_{xy}\right]}^{+}, \end{array}$$

(3) $$\begin{array}{c} {\text{LGN}}_{\text{OFF}}={\left[0.1-\underset{x}{\sum }\underset{y}{\sum }{\text{RF}}_{xy}{\text{PI}}_{xy}\right]}^{+}, \end{array}$$

where *σ*_center_=0.833 and *σ*_surr_=3*σ*_center_ (this yields the center width of 4 pixels and the RF diameter to ∼ 16 pixels.) are common space constants; *D*_*xy*_ is the distance between a pixel location (*x*, *y*) in the image and the RF center; LGN_ON_ and LGN_OFF_ are activity of ON-center and OFF-center LGN neuron, respectively; and finally, PI_*xy*_ is the grayscale pixel density at the (*x*, *y*) image location (0 ≤ PI_*xy*_ ≤ 1). ON- and OFF-center LGN neurons are placed on top of the window in a hexagonal form. On this window, 182 (91 ON-Center, 91 OFF-Center) LGN neuron outputs of the window are generated by passing a filter (similar to the high-pass filter) along the window. An example of thalamic output is shown in Figure 1.

## 4. Cortical Layer 4 Image Representation

**Figure 2:**
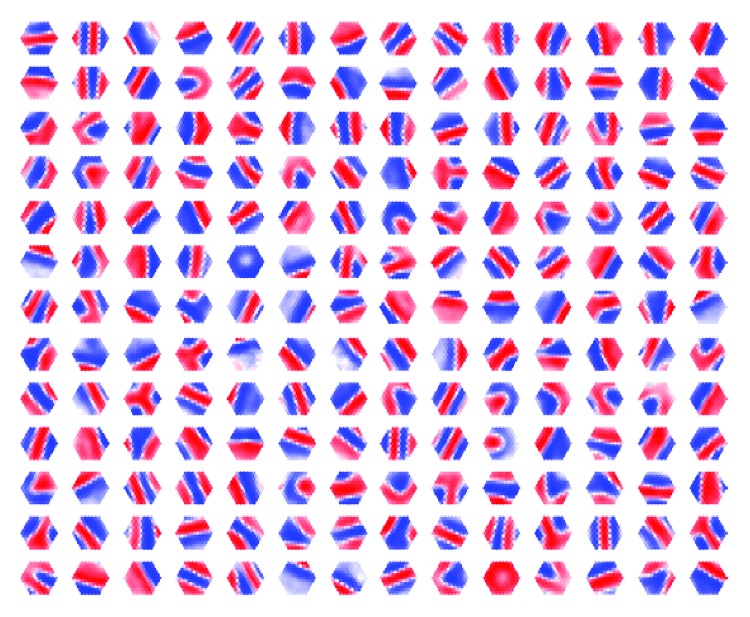
LGN patterns of all adapted 182 L4 cells in the trained network. The red color density indicates the power of the ON-center LGN activity, and the blue color intensity indicates the OFF-center LGN activity.

In the physiological structure, the filtered outputs from the thalamus are the inputs of L4 (Figure 2). Similarly, the mathematical model of the same structure is defined by a RBF-like feedback neuron model in [24]. The number of L4 function neuron outputs can be adjusted as desired. However, a total of 182 neurons have been selected in order to be comparable to the thalamus representation and to provide no advantage over the number of neurons:

(4) $$\begin{array}{c} \tau \frac{d}{dt}F_{i}=-F_{i}+\frac{1}{1-\theta }{\left[\overset{182}{\underset{j}{\sum }}w_{ij}{\text{LGN}}_{j}-\theta \sqrt{\overset{182}{\underset{j}{\sum }}{\text{LGN}}_{j}^{2}}+\lambda \overset{182}{\underset{k=1,k\neq 1}{\sum }}u_{ik}F_{k}\right]}^{+}, \end{array}$$

where *F*_*i*_ and *F*_*k*_ are the outputs of *i*th and *k*th L4 neurons; *τ* is a time constant (*τ*= 4 ms); *w*_*ij*_ is the weight of the *j*th RBF center; LGN_*j*_ is the activity of *j*th LGN cells; *θ* is the threshold value of the distance between the center of RBF and the excitation vector; *λ* is the scaling factor of lateral connections; and *u*_*ik*_ is the correlation coefficient between the output of *i*th and *k*th neurons of L4.

## 5. Natural Images

**Figure 3:**
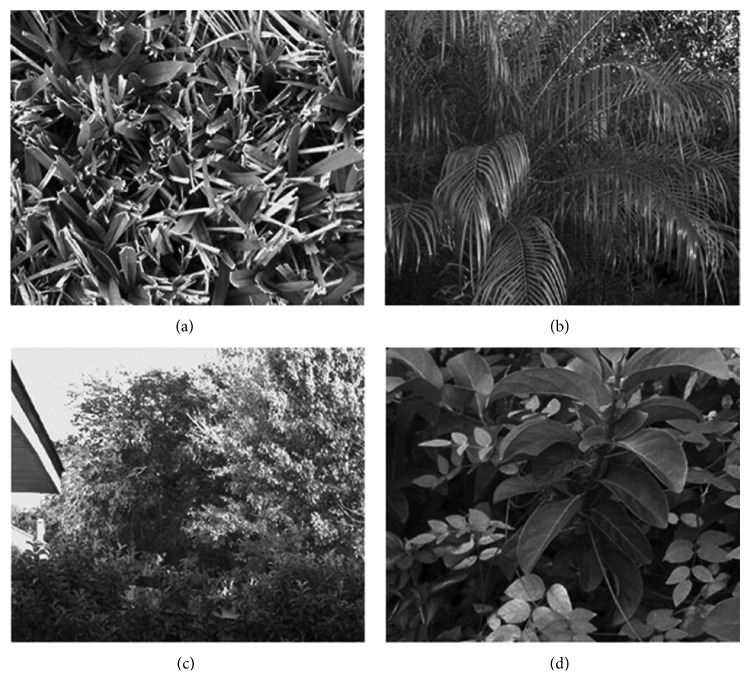
Four different image samples selected from a set of natural images used in the study.

Natural images are complex image clusters (e.g., mountain, landscape, forest, tree, house, meadow, etc.) which consist of many different pictures and contain patterns that are constantly experienced in daily life. In such models, the purpose of choosing natural images instead of artificial images is that the natural images and the inputs of human visual system are statistically similar. The visual inputs to the thalamus originate from grayscale images (a set of five 500 × 335 pixel images) containing natural patterns. The images were not preprocessed in any way (Figure 3).

## 6. Motion Blur Orientation

**Figure 4:**
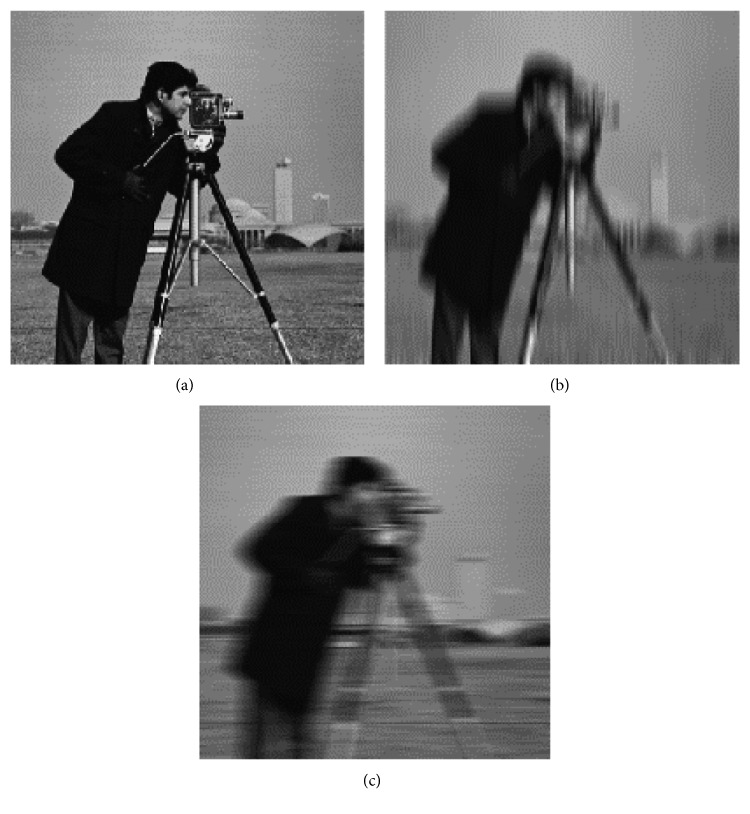
Motion blur orientation for a sample image. (a) Original image; (b) vertical motion; (c) horizontal motion.

Motion blur is the apparent striking of rapidly moving objects in a still image or a sequence of images (movie, animation, etc.). As the motion continues on the object, the examination of the objects seen from the outside or the objects in the outside world while moving causes the appearance of such motion traces [26]. From biological point of view, all the images he/she sees when in a live motion are affected by motion trajectory, making it difficult to detect the surrounding details. It is also a commonly used method in image processing methods for debluring process. The vertical (b) and horizontal (c) motion blur effects are applied on a sample image (a) as shown in Figure 4.

In visual cortex, vertically and horizontally adapted neurons can easily distinguish vertical (b) and horizontal (c) movements from the reference image. Especially with the increase of the motion blur size, the vertical or horizontal lines become more apparent, making it easier to determine in which direction the movement is. However, in the case of neurodegenerative diseases (Parkinson's disease, dementia, diabetic neuropathy, Alzheimer's, etc.) affecting the visual cortex, the direction in which the motion is applied cannot be perceived by patients, especially at very low motion blur size values (1–3 pixels).

From this hypothesis, the motion blur effect can be presented to the patients and controls by applying different directions and values to the natural image windows. It may also be possible to make inferences between individuals (patient vs control) according to test performances. However, it is necessary to first apply this to the mathematically modelled cortical models [24, 25] and then to compare them with the above mentioned groups. In this context, random window groups are selected from natural images, and then horizontal and vertical motion blur effects are applied to these windows with different pixel values. In this case, the performance of the linear SVM classifier [27] is measured by creating a problem of two classes (0: horizontal motion, 1: vertical motion).

## 7. Experimental Results

Based on the above information, a total of 4000 25 × 25 pixel sized windows were selected randomly from the natural images. Two different classes were created by creating horizontal motion and vertical motion classes of these selected images. Subsequently, the dataset was divided into two groups and assigned to the linear SVM classifier as training and test sets. The process is iterated 10 times, and average classifier performance ratios are calculated for every motion blur size values. In this case, the linear SVM is expected to classify the problem of vertical motion or horizontal motion on the incoming images. In addition, the performance of the classifier against the movements of different pixel values is also investigated in this study because the effect of the motion blur amount is also unknown.

**Figure 5:**
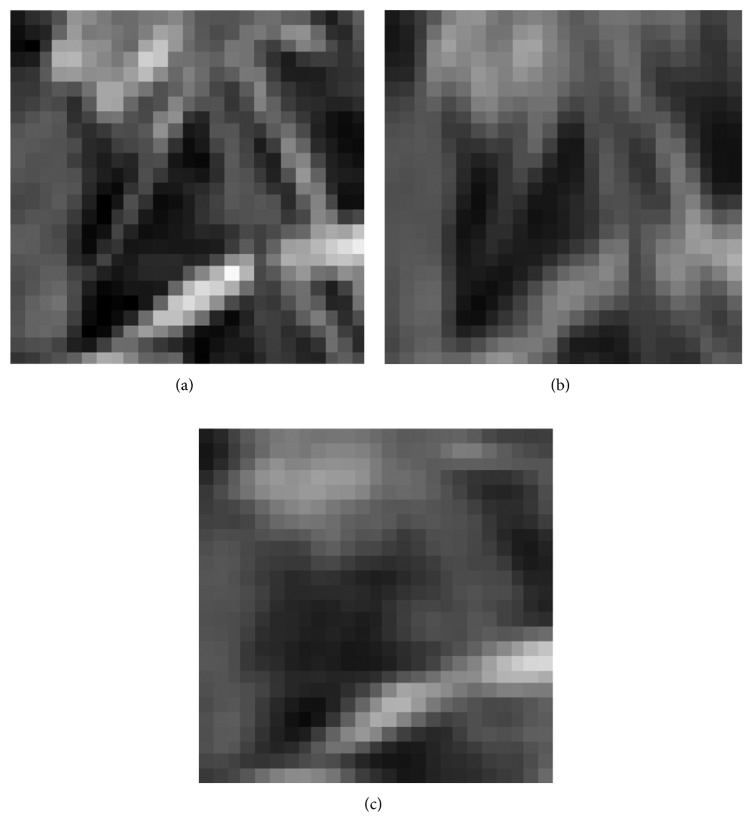
Motion blur applied on a natural image window (a), with vertical motion settings (b) and horizontal motion settings (c) (motion blur size: 5 pixels).

When Figures 4 and 5 are examined, it is seen that some patterns are preserved according to vertical and horizontal movement. If there is a vertical pattern in the image, such patterns become more apparent without being influenced by a vertical motion, but the horizontal patterns disappear according to the size of the motion blur. In the same way, a horizontal movement will cause horizontal patterns to become more apparent, resulting in the disappearance of vertical patterns.

**Figure 6:**
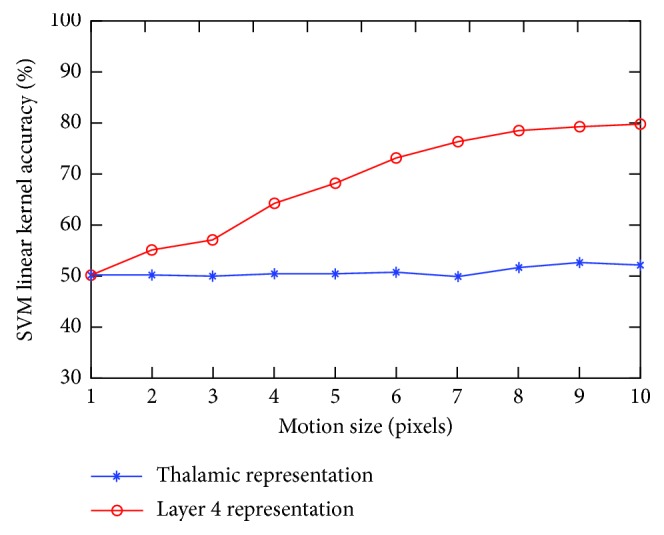
SVM linear classifier accuracy graph for motion blur test with different motion size values (pixels).

According to the obtained results, L4 representation has a performance of 78.38% in the vertical-horizontal motion blur distinction of 8 pixels selected as the reference value. However, the same value is 50% for the thalamic representation (Figure 6). It is observed that while the thalamic representation randomly classifies class labels, the L4 model classifies them according to a specific rule. The L4 model can classify the problem linearly according to the change values of the motion blur effect, while the thalamic representation cannot solve this problem with the linear classification methods, but another space transformation is needed.

**Figure 7:**
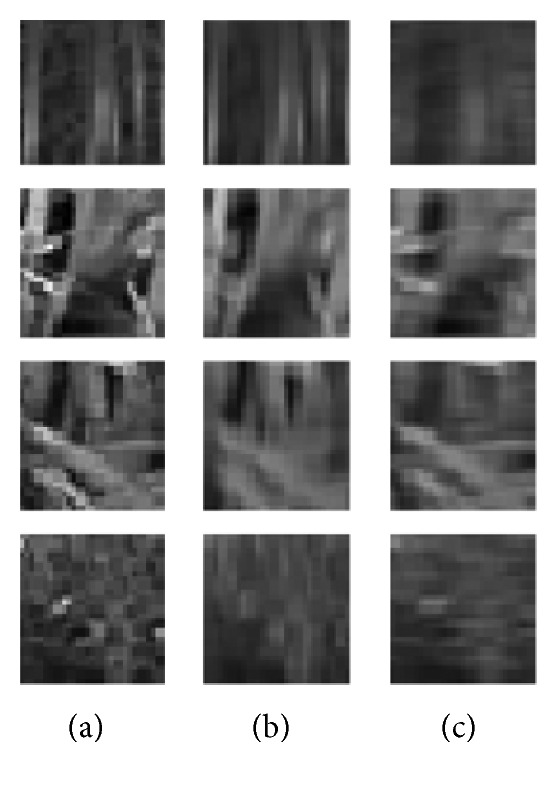
Misclassified randomly selected natural image windows: (a) original image window; (b) vertical motion blur; (c) horizontal motion blur.

**Figure 8:**
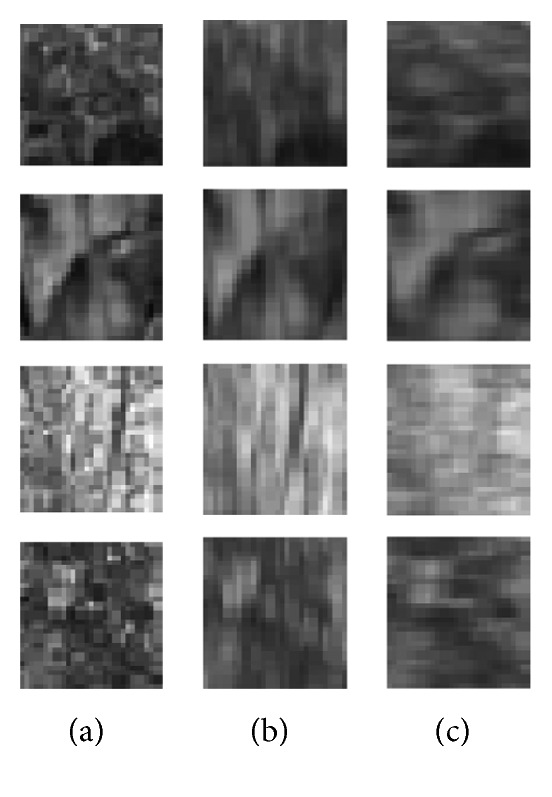
Correctly classified randomly selected natural image windows: (a) original image window; (b) vertical motion blur; (c) horizontal motion blur.

When randomly selected images that were classified correctly and incorrectly were examined for L4 representation, it was seen that the samples with little or no horizontal and vertical patterns were incorrectly classified (Figure 7) and but pattern-rich samples were correctly classified (Figure 8).

## 8. Discussion

In this study, a motion blur orientation-based visual test setup for Parkinson's disease has been proposed. By selecting random images, horizontal and vertical motion blur classification dataset has been build. This dataset is represented in mathematical models of L4 which is the first layer of visual cortex and thalamus which is the first chemoelectrical input filter of visual information. Then, these representations were given as inputs to the linear SVM classifier and were expected to solve the horizontal and vertical classification problem. Finally, L4 threshold values have been determined according to the linearly problem solution performance.

Neurons adapting to different patterns in the L4 model respond strongly to patterns similar to themselves. Images with horizontal and vertical patterns seem to be able to solve motion blur problems much more easily by adaptive L4 neurons which produce stronger outputs. The changes between the patterns in the increasing pixel movements can be solved linearly by Layer 4, whereas the thalamic structure requires a nonlinear transformation. The theory that Layer 4 transforms the images and projects a linearly solvable space is also proven with the test.

Favorov's work provides confirmatory results for the role of the pluripotential function linearization in cortical computation of L4. It has been shown that L4 has effective function linearization capabilities in the study, and it is shown that the upper layers can more easily compute complex functions such as classification and clustering problems with our tests.

When the L4 model is thought to be a physiology-based model, it is obvious that individuals with any neurodegenerative problem in the visual cortex will have difficulty in solving such problems. Classification performance of the ratio %80 can be regarded as a threshold value obtained by SVM classifier, so that if the purposed study is applied to the individuals, their scores are not to be expected higher than the threshold. With the provided test, healthy controls and patients with PD or other neurological disorders can be determined by examining their test scores.

## 9. Conclusion

PD is the second most common neurodegenerative disorder, and the mechanisms of neuronal degeneration visual cortex in PD are poorly known and there is no efficient visual test with physiological background. Our purposed cortex-based models and visual performance tests will help to discover this area.

In future studies, visual tests will be applied to the individuals to research for the answers of these two questions and aiming to compare the performances of powerful machine learning methods against the actual visual sensory scores. The same problem will be applied to both PD and control groups after the relevant ethical approvals are taken and scores will be deduced. Thus, inferences, such as spatial vision sensitivities of the disease and adaptation to color changes, can be determined by a noninvasive test.

## Data Availability

Image representations used to support the findings of this study are available from the corresponding author upon request.

## Supplementary Materials

Supplementary 1 File 1: classification of image representations with SVM machine learning classifier MATLAB source codes. Details are included in README file.

Supplementary 2 File 2: classification results in MATLAB figure format.

Supplementary 3 File 3: motion blur animation of an image to represent the problem.
