# Supplementary figures and images for: Cortical Visual Performance Test Setup for Parkinson's Disease Based on Motion Blur Orientation
Source: Parkinsons Dis. 2019 Feb 3;2019:3247608. doi: 10.1155/2019/3247608 (PMC6377996; doi:10.1155/2019/3247608)

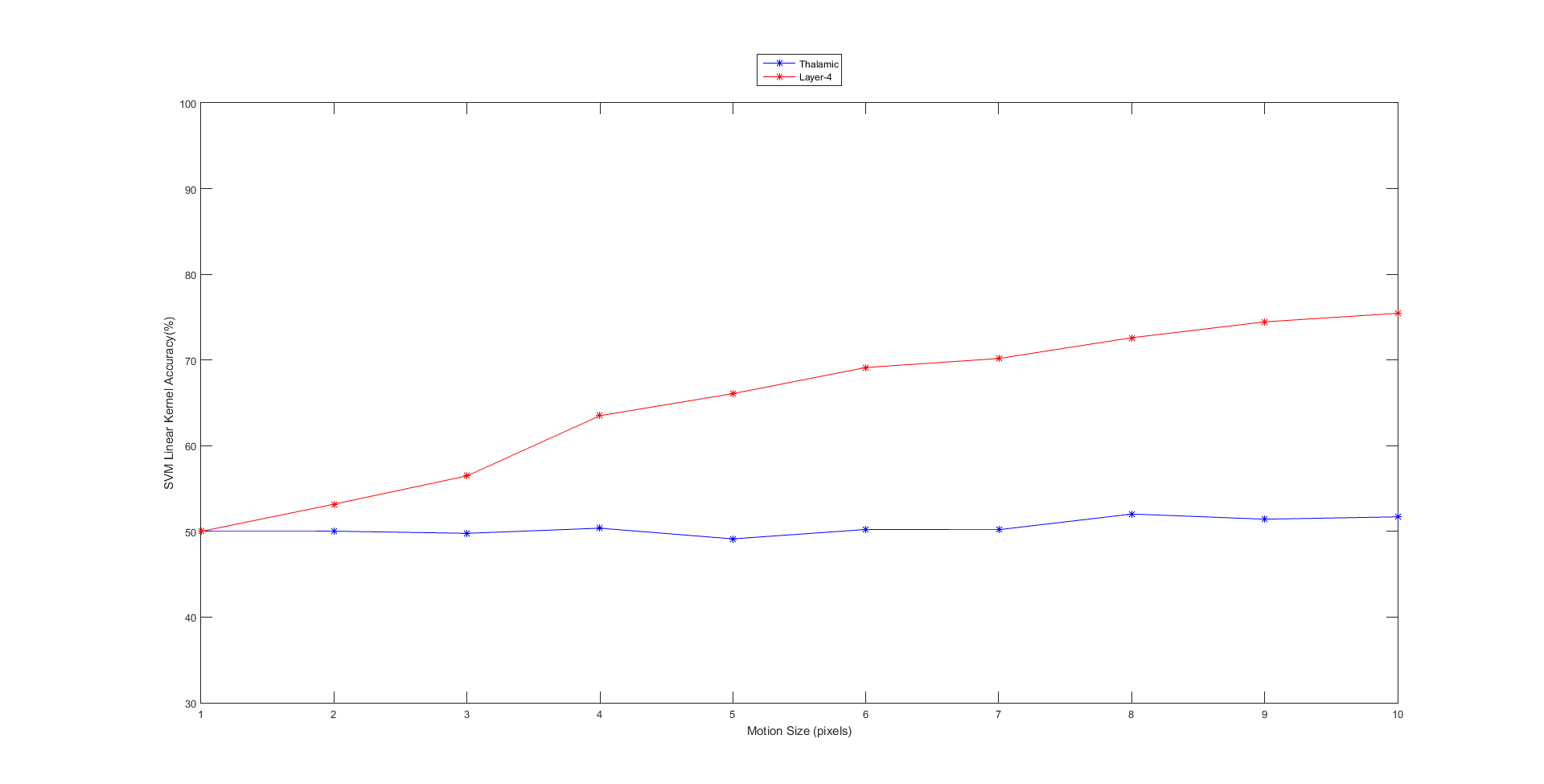

Supplement: Supplementary 1 — File 1: classification of image representations with SVM machine learning classifier MATLAB source codes. Details are included in README file. [file 3247608.f1.zip › 2-MatlabSource/results/classification_performance.tif]

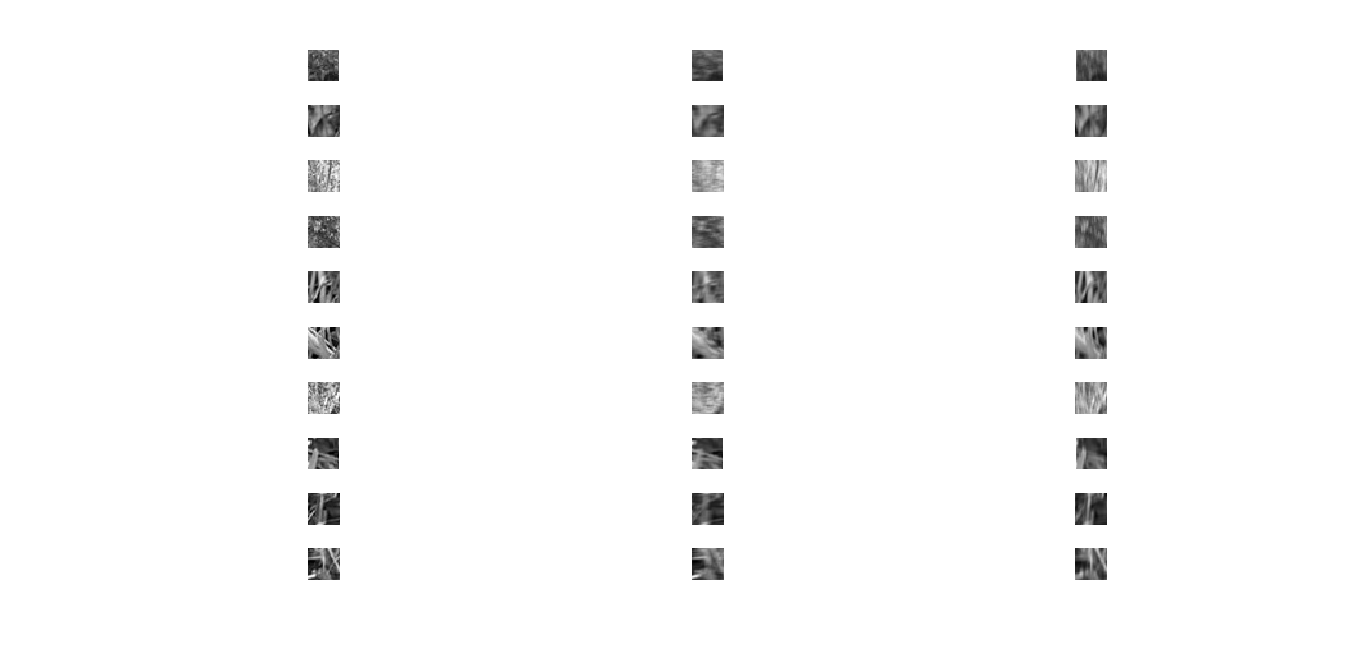

Supplement: Supplementary 1 — File 1: classification of image representations with SVM machine learning classifier MATLAB source codes. Details are included in README file. [file 3247608.f1.zip › 2-MatlabSource/results/high_wins.png]

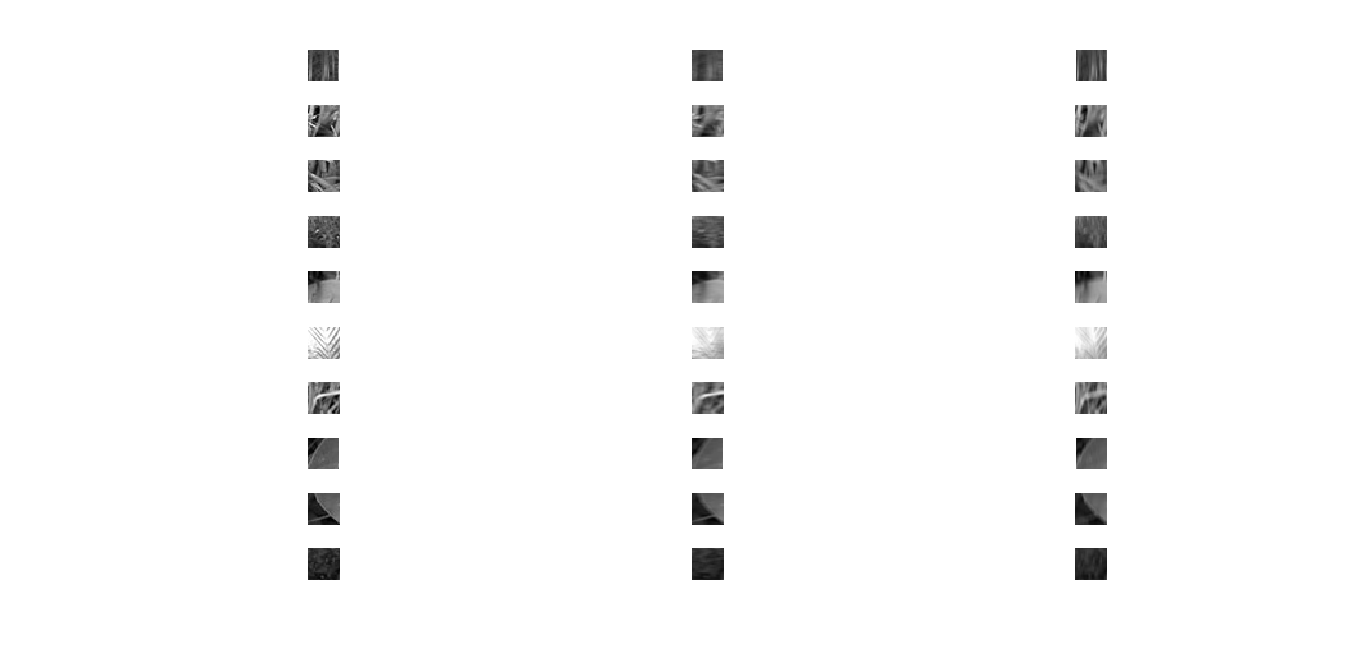

Supplement: Supplementary 1 — File 1: classification of image representations with SVM machine learning classifier MATLAB source codes. Details are included in README file. [file 3247608.f1.zip › 2-MatlabSource/results/low_wins.png]
